# Supplementary material for: The Systems Biology Research Tool: evolvable open-source software
Source: BMC Syst Biol. 2008 Jun 29;2:55. doi: 10.1186/1752-0509-2-55 (PMC2446383; doi:10.1186/1752-0509-2-55)
Supplement: Additional file 1 — SBRT Archive. An archive of the current version of the Systems Biology Research Tool. [file 1752-0509-2-55-S1.zip › sbrt-1.4.0/doc/developers_guide/api/sbrt/kernel/fba/proc/ExtremeCurrentProcess.html]

ExtremeCurrentProcess


|  |  |  |
| --- | --- | --- |
| **Overview** \| **Package** \| **Class** \| **Use** \| **Tree** \| **Deprecated** \| **Index** \| **Help** | |  |
| **PREV CLASS**   **NEXT CLASS** | **FRAMES**    **NO FRAMES**     **All Classes** |  |
| SUMMARY: NESTED \| FIELD \| CONSTR \| METHOD | DETAIL: FIELD \| CONSTR \| METHOD |  |


---


## sbrt.kernel.fba.proc Class ExtremeCurrentProcess

```
java.lang.Object
  sbrt.kernel.fba.proc.FbaProcess<DoubleMatrix<?,java.lang.String>>
      sbrt.kernel.fba.proc.ExtremeCurrentProcess
```

**All Implemented Interfaces:**: KernelProcess<DoubleMatrix<?,java.lang.String>>, MonitoredProcess<DoubleMatrix<?,java.lang.String>>

---

``` public final class ExtremeCurrentProcess extends FbaProcess<DoubleMatrix<?,java.lang.String>> implements MonitoredProcess<DoubleMatrix<?,java.lang.String>> ```

This class is used to perform extreme current experiments.

**Author:**
:   This class was written and documented by Jeremiah Wright
    while in the Wagner lab.

---

**Constructor Summary**

`ExtremeCurrentProcess(IrrevRxnFluxome fluxome,
ExtremeCurrentComputer computer)`
  
          Constructs a new extreme current experiment.


| **Method Summary** | |
| --- | --- |
| `IrrevRxnFluxome` | `getFluxome()`             Returns the fluxome used in this experiment. |
| `ProgressMonitor` | `getProgress()`             Returns a progress monitor for this experiment. |
| `void` | `run(ResultsManager<DoubleMatrix<?,java.lang.String>> resultsManager)`             Runs this process and adds the generated results to the provided results manager via its `ResultsManager.addData(Object)` method. |

**Methods inherited from class sbrt.kernel.fba.proc.FbaProcess**

`toString`

**Methods inherited from class java.lang.Object**

`clone, equals, finalize, getClass, hashCode, notify, notifyAll, wait, wait, wait`

**Constructor Detail**

### ExtremeCurrentProcess

```
public ExtremeCurrentProcess(IrrevRxnFluxome fluxome,
                             ExtremeCurrentComputer computer)
```

:   Constructs a new extreme current experiment.

    **Parameters:**: `fluxome` - the fluxome whose extreme currents are to be computed.: `computer` - the computer with which all results will be computed.


**Method Detail**

### getFluxome

```
public IrrevRxnFluxome getFluxome()
```

:   Returns the fluxome used in this experiment.

    :   **Specified by:**: `getFluxome` in class `FbaProcess<DoubleMatrix<?,java.lang.String>>`
    :   **Returns:**: the fluxome used in this experiment.

---


### getProgress

```
public ProgressMonitor getProgress()
```

:   Returns a progress monitor for this experiment.

    :   **Specified by:**: `getProgress` in interface `MonitoredProcess<DoubleMatrix<?,java.lang.String>>`
    :   **Returns:**: a progress monitor for this experiment.

---


### run

```
public void run(ResultsManager<DoubleMatrix<?,java.lang.String>> resultsManager)
```

:   Runs this process and adds the generated
    results to the provided results manager via
    its `ResultsManager.addData(Object)`
    method.

    :   **Specified by:**: `run` in interface `KernelProcess<DoubleMatrix<?,java.lang.String>>`
    :   **Parameters:**: `resultsManager` - the results manager to which the results will be added.


---


|  |  |  |
| --- | --- | --- |
| **Overview** \| **Package** \| **Class** \| **Use** \| **Tree** \| **Deprecated** \| **Index** \| **Help** | |  |
| **PREV CLASS**   **NEXT CLASS** | **FRAMES**    **NO FRAMES**     **All Classes** |  |
| SUMMARY: NESTED \| FIELD \| CONSTR \| METHOD | DETAIL: FIELD \| CONSTR \| METHOD |  |


---
